# Exploring Indonesian actinomycete extracts for anti-tubercular compounds: Integrating inhibition assessment, genomic analysis, and prediction of its target by molecular docking

**DOI:** 10.1016/j.heliyon.2024.e35648

**Published:** 2024-08-04

**Authors:** Arif Nurkanto, Masrukhin, Joseph Calvin Erdian Tampubolon, Muhammad Farrel Ewaldo, Ade Lia Putri, Shanti Ratnakomala, Ruby Setiawan, Ahmad Fathoni, Kartika Dyah Palupi, Yulia Rahmawati, Danang Waluyo, Erwahyuni Endang Prabandari, Sri Pujiyanto, Yuji Sumii, Andria Agusta, Norio Shibata, Sohkichi Matsumoto, Tomoyoshi Nozaki

**Affiliations:** a Research Center for Biosystematics and Evolution, Research Organization for Life Sciences and Environmental, National Research and Innovation Agency (BRIN), West Java, Indonesia; b Graduate School of Medicine, The University of Tokyo, Tokyo, Japan; c Department of Biology, Faculty of Science and Mathematics, Diponegoro University, Central Java, Indonesia; d Master's Programme in Biomedical Science, Faculty of Medicine, University of Indonesia, West Java, Indonesia; e Research Center for Pharmaceutical Ingredients and Traditional Medicine, National Research and Innovation Agency (BRIN), West Java, Indonesia; f Research Center for Vaccine and Drug, Research Organization for Health, National Research and Innovation Agency (BRIN), Banten, Indonesia; g Department of Frontier Materials, Nagoya Institute of Technology, Nagoya, Japan; h Department of Bacteriology, School of Medicine, Niigata University, Niigata, Japan; i Laboratory of Tuberculosis, Institute of Tropical Disease, University of Airlangga, Surabaya, East Java, Indonesia; j Division of Research Aids, Hokkaido University Institute for Vaccine Research & Development, Sapporo, Japan

**Keywords:** Tuberculosis, Actinomycetes, Screening, Extracts, Active compound, Whole genome, Docking study, Shikimate kinase

## Abstract

Tuberculosis (TB) is the foremost cause of infectious fatality globally. The primary global challenge in combatting TB lies in addressing the emergence of drug-resistant variants of the disease. However, the number of newly approved agents for treating TB has remained remarkably low over recent decades. Hence, research endeavors for discovering novel anti-TB agents are always needed. In the present study, we screened over 1,500 culture extracts from actinomycetes isolated in Indonesia for their inhibitory activity against *Mycobacterium smegmatis* used as a surrogate in the primary screening. The initial screening yielded approximately 6.2 % hit extracts, with a selection criterion of >80 % growth inhibition. The confirmed hit extracts were subsequently subjected to growth inhibition assay against *Mycobacterium bovis* and *Mycobacterium tuberculosis.* Approximately 20 % of the hit extracts that showed growth inhibition also exhibited efficacy against *M. bovis* BCG and *M. tuberculosis* H37Rv pathogenic strain*.* An active compound was successfully purified from a large-scale culture of the most potent representative extract by high-performance liquid chromatography and thin-layer chromatography. The structure of the active compound was elucidated by mass spectrometry and nuclear magnetic resonance. This compound displayed structural similarities to actinomycin group and exhibited robust inhibition, with IC_50_ values of 0.74, 0.02, and 0.07 μg/mL against *M. smegmatis*, *M. bovis,* and *M. tuberculosis*, respectively. The Actinomycetes strain A612, which produced the active compound, was taxonomically classified by phylogenetic analysis of 16s rRNA gene and whole genome sequencing data as *Streptomyces parvus*. Computational genome analysis utilizing anti-SMASH 7.0 unveiled that *S. parvus* A612 strain harbors 40 biosynthetic gene clusters with the potential to produce 16 known (with >70 % similarity) and 24 unknown compounds. A non-ribosomal peptide synthesis (NRPS) gene cluster associated with actinomycin D biosynthesis was also identified, boasting an 85 % similarity. Molecular docking analysis of actinomycin D and 21 potential *M. tuberculosis* targets revealed possible interactions with multiple targets. The purified active compound inhibited recombinant *M. tuberculosis* shikimate kinase (*Mt*SK), which validated the results obtained from the docking analysis.

## 1 Introduction

Tuberculosis (TB) continues to persist as a pressing global issue within the field of infectious diseases. The causative agents of TB belong to the *Mycobacterium tuberculosis* complex, along with its African variants (*Mycobacterium africanum* and *Mycobacterium canettii*) and *Mycobacterium bovis* [1]. TB remains a formidable global public health challenge, with a staggering 10.6 million cases and 1.6 million deaths recorded worldwide in 2021 alone. These figures mark an escalation from the respective tallies of 1.5 million cases and 1.4 million deaths reported in 2019 and 2020 [2].

Adding to the gravity of the situation is the upward trajectory of individuals affected by drug-resistant strains, which poses a significant peril to global health. The latest data from the World Health Organization (WHO) is disconcerting, projecting around 465,000 new cases and 182,000 deaths resulting from MDR- (multi-drug resistant) or RIF-resistant-TB in 2019 [3]. Presently, the most pressing challenge that the scientific community faces concerning TB on a global scale is combating the emergence of drug-resistant variations of the disease, particularly the formidable foes of multidrug-resistant TB (MDR-TB) and extensively drug-resistant TB (XDR-TB). A paramount concern is the emergence of Totally Drug-Resistant Tuberculosis (TDR-TB), which has been reported in some countries [4,5].

Annually, numerous newly identified molecules are hailed as promising agents in the fight against TB, however, over the past 40 years, only three novel agents (bedaquiline, delamanid, pretomanid) have successfully obtained marketing authorization as anti-TB drugs [6]. On a positive note, recent information from the Working Group for New TB Drugs (WGND) indicates a glimmer of hope in the global pipeline of tuberculosis drugs. Approximately 23 drug candidates are currently progressing through diverse stages of clinical development, Phase 1–3 [7].

Based on the data and facts presented, drug discovery to find novel compounds for TB treatment remains highly important and necessary. Natural products play a pivotal role in the field of drug discovery. They account for over 75 % of the antibiotics available [8], with a significant contribution from bacteria, particularly within the Streptomyces genus. In fact, bacteria from the Streptomyces genus are responsible for producing over two-thirds of all therapeutically effective antibiotics [9,10]. Actinomycetes collectively produce over 10,000 compounds, with Streptomyces being a major contributor by generating more than 7,600 of these compounds. Several clinically effective antitubercular drugs, including streptomycin, rifampicin, kanamycin, and capreomycin, were derived from actinomycetes through isolation and development processes. These compounds have played a crucial role in the treatment of TB for many years and continue to be important components of current TB treatment regimens. Some novel compounds with anti TB activity were also discovered such as caprazamycin B [11], ecumicin [12], chrysomycin A [13], and some analogs of azomycin [14,15]. Hence, there is an ongoing need to discover new anti-TB compounds from actinomycetes.

The pace of advancement in drug discovery against *M. tuberculosis* is hindered by several technical challenges. This pathogen is a slow-growing bacteria, primarily affecting the respiratory system, and requires handling in Biosafety Level 3 (BSL3) facilities. Furthermore, the process of TB drug discovery faces additional challenges. These include the formidable barrier posed by the low permeability of the cell wall, the presence of active multi-drug efflux pumps, and the existence of a heterogeneous population within the host. These factors collectively contribute to the limited efficacy of many anti-TB drugs.

Various approaches have been developed to acquire anti-TB compounds, with a particular emphasis on minimizing time and pathogenic effects. To date, phenotypic screening has demonstrated greater potential compared to target-based approach [16]. One such approach is phenotypic screening using a *mycobacterium* model. In many instances, early and even some current efforts in anti-TB drug discovery have employed *M. bovis* (BCG) or *Mycobacterium smegmatis* as principal bacteria for phenotypic screens [16]. While using *M. smegmatis* or *M. bovis* as surrogates for pathogenic bacteria aids in circumventing slow growth and safety concerns, it also introduces the risk of overlooking potential anti-TB candidates that specifically killed the pathogenic bacteria but not the non-pathogenic one. This approach is substantiated by prior findings indicating that rapid screening using non-pathogenic mycobacteria has the potential to unveil effective anti-TB compounds. For example, the discovery of bedaquiline (TMC207), a diarylquinoline, resulted from a screen against *M. smegmatis* [17]. This demonstrates that such surrogate mycobacteria could be employed to overcome challenges associated with slow growth and safety considerations in the screening process.

In this study, we present our investigation of actinomycete extracts through screenings against *M. smegmatis*. Hits were selected based on their inhibition levels exceeding 80 %, which were then further validated using *M. bovis* and the pathogenic strain *M. tuberculosis* H37Rv. The extracts that exhibited efficacy against *M. tuberculosis* were chosen for large-scale extract production and subsequent purification of active compounds, followed by identification through NMR and MS techniques. The strain responsible for producing these active compounds was also identified using the 16S rRNA gene. Additionally, a comprehensive whole-genome analysis was conducted to identify gene clusters and gain insights into potential secondary metabolites produced by this strain. To enhance our comprehension of the mechanism behind the active compounds, we conducted docking studies against selected protein drug targets in *M. tuberculosis*.

## 2 Materials and methods

### 2.1 Bacterial strains and culture conditions

*M. smegmatis* mc^2^155, *M. bovis* BCG Pasteur, and *M. tuberculosis* H37Rv were cultivated in Middlebrook 7H9 broth (Sigma-Aldrich) with 0.5 % glycerol, 0.05 % Tween 80 and supplemented with 10 % OADC, comprising 0.06 % oleic acid, 5 % bovine serum albumin (BSA), 2 % dextrose, and 0.85 % sodium chloride. The bacterial cultures were incubated at 37 °C with shaking at 200 rpm.

### 2.2 Actinomycetes isolates, cultures and extract production

Primary screening was performed using microbial extracts from actinomycetes. The actinomycetes were collected from soil, sediment, and leaf litter from several locations in Indonesia, including the Karimun-Riau Islands, Palembang-South Sumatra, East Kalimantan, and Kupang-East Nusa Tenggara province. Actinomycetes isolates were cultured in Yeast Starch Broth (YSB) medium in 250-mL flasks and placed in a rotary shaker incubator at 220 rpm and 28 °C for 7 days. Following incubation, an equal amount of ethyl acetate was added and mixed for 1 h. The upper organic phase was then obtained and evaporated in a 40 °C water bath. The crude extracts were solubilized using 50 % DMSO prior to their use in primary screening.

### 2.3 Actinomycetes screens for anti-mycobacterial activity

Screening was performed using a Resazurin reduction assay according to the previous method [18], actinomycetes extracts were screened for inhibition of mycobacterial growth. The mycobacterial strains were grown to the mid-logarithmic phase and diluted to an OD_600_ of 0.05. To minimize evaporation during assay incubation, 100 μl of sterile-distilled water was added to the wells in the first column of a 96-well plate. A 49 μl of growth media was transferred to columns 2 to 11 of the assay plates. One μl of each extract from the stock plates was transferred to the wells in columns 2 to 11 using a multi-channel pipette to obtain a final concentration of 100 μg/mL extracts (0.5 % DMSO) in each well. Media, bacterial suspension, and DMSO were dispensed appropriately in column 12 of each plate as controls. 50 μl of the diluted *M. smegmatis* culture was added to all wells except the media control wells. The assay plates were sealed, wrapped in parafilm, and incubated at 18 h at 37 °C. A 40 μL solution of Resazurin (20 mg/100 mL diluted 1:1 with 10 % Tween 80) was added and incubated further for an additional 12 h at 37 °C for color development before being observed using a fluorescence microplate reader (Varioskan Lux, Thermo Fisher Scientific, USA) with excitation 540 nm and emission 590 nm. The actinomycetes extract screens were performed in three independent experiments, and the data were compiled and analyzed in Excel to identify compounds that exhibited ≥80 % growth inhibition. Z′-factor and signal-to-background ratio (S/B) were calculated to assess the quality of the screening system, following previously described methods [19].

Samples indicating ≥80 % inhibition against *M. smegmatis* were subsequently tested for their inhibitory activity against *M. bovis* and *M. tuberculosis* H37Rv. Extracts demonstrating the highest and comparable inhibition against these three mycobacterium strains (*M. smegmatis*, *M. bovis* and *M. tuberculosis*) were selected for further analysis, such as compound purification and identification, half-maximal inhibitory concentration (IC_50_) determination, and cytotoxicity assay.

### 2.4 Cultivation and metabolite extraction from selected active strain

The pre-culture was grown in 10 ml of YSB in 100 mL Erlenmeyer flasks for three days at 28 °C on rotary shakers at 180 rpm agitation. Subsequently, pre-culture was inoculated to the primary cultures (2 L of YSB medium in 5 L flasks) cultivated at 28 °C on a rotary shaker at 180 rpm for seven days. Metabolites were extracted with an equal volume of ethyl acetate and evaporated.

### 2.5 Chromatography-based purification of the active compound

The crude extract was observed by thin-layer chromatography (TLC) on a TLC sheet. An optimized solvent system was performed using TLC before applying it to silica in open-column chromatography. The sample was further separated by open column chromatography using silica gel 60 with a mesh size of 230–400 (Merck) as the stationary phase. In this purification process, 237.5 mg crude extract was loaded onto the open column chromatography. The eluent used was a mixture of dichloromethane and methanol (20:1) solvent system from the previous optimization. The eluate was collected and evaporated, and then each fraction was analyzed by bioassay. Fractions containing single and identical compound spots showing anti-mycobacterial activity were combined and concentrated. The combined fraction displaying anti-mycobacterial activity was then re-purified using the same open column mentioned earlier to obtain a pure active compound. To attain the highest level of purity, we implemented an additional two-step column purification procedure by increasing the length of the column, reducing its diameter, and using a finer silica gel mesh size.

The final-purified active fraction was evaluated by reverse phase high-performance liquid chromatography (HPLC) analysis employing a mobile phase composed of acetonitrile (A) and water (B). The gradient profile was optimized as follows: 0–10 min, isocratic 40 % (v/v) A; 10–25 min, linear gradient 41–80 % (v/v) A; 26–35 min, isocratic 80 % (v/v) A. The flow rate was maintained at 1 ml/min, and the column compartment was maintained at a temperature of 30 °C. Two 255 nm and 366 nm detection wavelengths were simultaneously employed to record chromatograms. The injection volume was 35 μL and the compound concentration was 10 mg/mL. Only active compound with a purity exceeding 97 % was used for chemical structure elucidation.

### 2.6 Structural elucidation of active compound

The mass spectra (MS) analysis was carried out using the UPLC–qTOF-MS/MS system (Waters). The analysis involved a flow rate of 0.3 mL/min, a temperature set at 40 °C using a C_18_ column (Agilent, 2.1 mm × 50 mm, 1.7 μm). The mobile phase consisted of 0.1 % formic acid in H_2_O (solvent A) and 0.1 % formic acid in acetonitrile (solvent B). The isocratic elution system was initiated with a ratio of A:B at 95:5 from 0 to 1.0 min, followed by a linear gradient increase in solvent B from 5 % to 40 % (from 1.0 to 8.0 min), then a linear gradient increase from 40 % to 100 % solvent B (from 8.0 to 11.00 min). The isocratic elution system was maintained with 100 % eluent B from 11 to 13 min, followed by a linear gradient decrease of solvent B from 100 % to 5 % (from 13 to 16 min). For the MS/MS analysis, the ESI interface was used in positive mode with the following settings: source temperature at 120 °C, capillary voltage at 2 kV, desolvation temperature at 500 °C, desolvation gas flow at 1000 L/h, and conical gas flow at 50 L/h. The structure of the compound was determined using nuclear magnetic resonance (NMR) spectroscopy performed on a JOEL ECA-500 spectrometer (Japan) equipped with a 2.5-mm microprobe. CDCl3 served as the solvent for the ^1^H NMR experiment, and the spectrum was recorded at 23 °C. ^1^H NMR spectra were recorded in CDCl3 at 500 MHz, and the chemical shifts were reported in parts per million (ppm) with coupling constants in Hz. The chemical shifts were referenced relative to the solvent peaks (CDCl3: 1H δ 7.24).

### 2.7 Dose-response assays for half-maximal inhibitory concentration (IC_50_) determination

Cultures of *M. smegmatis*, *M. bovis*, and *M. tuberculosis* were grown to the mid-logarithmic phase and diluted to an optical density at 600 nm of 0.05. In triplicate, 50 μl of 7H9 broth containing purified compound, control antibiotics, and control solvent were added to the wells. Two-fold serial dilutions were performed to the indicated well (final concentration ranging from 0.4 to 50 μg/mL). 50 ml of the diluted cell culture was then added to the wells. Wells containing cell cultures without antibiotics were used as growth controls, and no cell cultures were added to the media control wells. The assay plates were sealed and incubated at 37 °C. Growth inhibition was monitored using a Resazurin reduction assay described previously. The compounds were tested in triplicate, and their half-maximal inhibitory concentration (IC_50_) values were determined using GraphPad Software (San Diego, California, USA).

### 2.8 Cytotoxicity assay of active compound

The cytotoxicity assay involved the evaluation of cytotoxic effects on human cells using the colorectal adenocarcinoma cell line (DLD-1). The DLD-1 cells were cultured in a 75 cm^2^ flask (Violamo) at 37 °C, with Roswell Park Memorial Institute (RPMI 1640) medium supplemented with 10 % FBS, 2.1 mM stable Glutamine, and 2.0 g/L NaHCO_3_. DLD-1 cells in a semiconfluent state were detached from the flask by incubating them with 5 mL of RPMI 1640 containing 0.25 % trypsin-EDTA to conduct the assay. After detachment, the DLD-1 cells were resuspended in RPMI 1640 supplemented with FBS, l-glutamine, and NaHCO_3_, and their cell viability was assessed using 0.4 % Trypan blue on a hemocytometer. Approximately 100 μL of the cell suspension, containing 3,000 cells, was added to each well of a 96-well clear-bottom plate. Cells were dispensed into the wells containing 2 μL of compounds dissolved in 50 % DMSO (final compound concentrations: 100, 20, 4, 0.8, and 0.2 μM and final DMSO concentration was 1 % each well). The culture plate was then incubated at 37 °C under 5 % CO_2_. Following 48 h of cultivation, 10 μL of Cell Counting Kit 8 (Dojindo, Japan) was added to each well, and the plates were further incubated for 2 h to examine cell growth and survival. The absorbance at 450 nm wavelength was measured using a microplate reader. The assay was conducted in triplicate for each dilution.

### 2.9 Molecular docking utilizing the structure of actinomycin D as the active compound

X-ray crystal structures of multiple protein targets that play a pivotal role in activating *M. tuberculosis* pathogens were utilized. Protein data were acquired from the Research Collaboratory for Structural Bioinformatics Protein Data Bank (RCSB PDB, http://www.rcsb.org/pdb/home/home.do). We selected 21 enzymes target as follow: *Mt* enoyl reductase (InhA; *Mt*InhA; PDB ID: 4TRJ), *Mt* Coenzyme A biosynthesis bifunctional protein (CoaBC; *Mt*CoaBC; Uniprot ID: P9WNZ1), *Mt* Shikimate Kinase (SK) bindingsite-1(*Mt*SK-BD1; PDB ID: 2IYW), *Mt* Shikimate Kinase (SK) bindingsite-2 (*Mt*SK-BD2, PDB ID: 2IYW), *Mt* Pantothenate kinase (PanK; *Mt*PanK; PDB ID: 5XLV), *Mt* 3-oxoacyl-[acyl-carrier-protein] reductase (MabA; *Mt*MabA; PDB ID: 1UZN), *Mt* 4-hydroxy-tetrahydrodipicolinate reductase (DHPR; *Mt*DHPR; PDB ID: 5TJZ), *Mt* dihydrofolate reductase (DHFR; *Mt*DHFR; PDB ID: 6NNH), *Mt* Sterol 14alpha-demethylase (CYP51; *Mt*CYP51; PDB ID: 1 × 8V), *Mt* 1-deoxy-d-xylulose 5-phosphate reductoisomerase (DXR; *Mt*DXR; PDB ID: 4A03), *Mt* Aspartate-semialdehyde dehydrogenase (ASDH; *Mt*ASDH; PDB ID: 3VOS), *Mt* decaprenylphosphoryl-ß-d-ribose-2′-oxidase (DprE1, *Mt*DprE1; PDB ID: 6HFV), *Mt* 2-dehydropantoate 2-reductase (PanE; *Mt*PanE; PDB ID: 4OL9), *Mt* Mycocyclosin synthase (CYP121; *Mt*CYP121; PDB ID: 7S0O), *Mt* Adenosylmethionine-8-amino-7-oxononanoate aminotransferase (BioA; *Mt*BioA; PDB ID: 4WYD), *Mt* Polyketide Synthase 13-Thioesterase (Pks13-TE; *Mt*Pks13-TE; PDB ID: 7VJT), *Mt* antigen 85 (Ag85) complex (*Mt*Ag85C; PDB ID: 5KWI), *Mt* biotin protein ligase (*Mt*BPL; PDB ID: 3RUX), *Mt* malate synthase (*Mt*MS; PDB ID: 6DL9), *Mt* 1,4-Dihydroxy-2-naphthoyl-CoA synthase (MenB; *Mt*MenB; PDB ID: 4QII), and *Mt* Citrate lyase beta-subunit (CitE; *Mt*CitE; PDB ID: 6AQ4). The preparation of receptors involved the utilization of Biovia Discovery Studio Visualizer 2020 and MGLTools 1.5.6 [20].

The co-crystallized ligands, water molecules, and cofactors within each receptor were removed, and Gasteiger charges were introduced individually. The two-dimensional chemical structure of the active compound (actinomycin D) was initially drawn using ChemDraw Ultra and then converted to its three-dimensional coordinates using the Chem3D Ultra program. The resulting structures that underwent energy minimization through the MM2 method were saved in PDB format. Additionally, non-polar hydrogen atoms within the ligands were combined, and rotatable bonds were defined using MGL Tools 1.5.6.

Molecular docking simulations were conducted using AutoDock Vina 1.1.2 with the default protocol, and the exhaustiveness was set to 12 [21]. A grid box with dimensions of 30 points in all directions was established for each receptor, with a grid spacing of 1 Å centered around the respective co-crystallized ligands. Following the docking computations, the most favorable poses were selected from the top ten models for each target based on evaluations of their binding energy (ΔG binding, kcal/mol) and non-bond interactions profile [22]. Subsequent analyses of molecular interactions were carried out using Biovia Discovery Studio Visualizer 2020 and PyMoL 1.7.4 software programs.

### 2.10 Revalidating docking results through the *M. tuberculosis* enzyme assay

In this study, we conducted enzymatic assays to assess the inhibition of the pure compound isolated in this experiment. We also assayed control and standard drugs (rifampicin and actinomycin D). At this time, we focused on three essential enzymes in *M. tuberculosis*: Shikimate kinase (*Mt*SK), phosphopantothenoylcysteine synthetase phosphopantothenoylcysteine decarboxylase (*Mt*CoABC), and 5-enolpyruvylshikimate-3-phosphate synthase (*Mt*EPSPS), based on docking results. All these enzymes were prepared as recombinant proteins expressed in *E. coli* (details not included in this study or unpublished data). For the *Mt*SK inhibition assessment, we quantified the production of ADP in the reaction using a coupling assay, as previously described [23]. In the case of *Mt*CoABC and *Mt*EPSPS inhibition, we employed a pyrophosphate assay with the BIOMOL® Green reagent (Enzo Life Sciences) [24]. All assays were conducted on a 96-well plate format, with each well having a final volume of 50 μL. The inhibition levels were calculated as a percentage of inhibition for each compound, relative to the activity observed with only DMSO as the control (no inhibition), with no enzyme present, set as 100 % inhibition. A range of compound dilutions was utilized, with final concentrations of 160, 80, 40, 20, 10, 5, 2.5, and 1.25 μg/mL, to determine IC_50_ values. The assay was conducted in triplicate for each dilution, and IC_50_ values were calculated using GraphPad Software (San Diego, California USA).

### 2.11 Molecular identification and phylogenetic construction of actinomycetes producing anti-mycobacterial compound

The DNA was extracted using PrepMan Ultra Reagent (Applied Biosystems, Foster City, CA, USA) following the manufacturer's instructions. For PCR amplification of the 16S rRNA gene, a pair of primers, 9F (5′-GAGTTTGATCCTGGCTCAG-3′) and 1541R (5′-AAGGAGGTGATCCAGCC-3′), was utilized. The resulting PCR product was sequenced using an automated DNA sequencer (ABI PRISM 3730 Genetic Analyzer, Applied Biosystems). To determine the nearly complete sequence of the 16S rRNA gene, four primers, namely 9F, 785F (5′-GGATTAGATACCCTGGTAGTC-3′), 802R (5′-TACCAGGGTATCTAATCC-3′), and 1541R, were employed. The EzTaxon-e server was used to calculate the similarities among the 16S rRNA gene sequences. The alignment of the 16S rRNA gene sequences of strain and other related genus members was performed using the CLUSTAL_X program. Phylogenetic trees were reconstructed using the neighbor-joining, maximum-likelihood, and maximum-parsimony algorithms. The resulting tree topology was assessed using bootstrap analysis with 1,000 replicates.

### 2.12 Genomic analysis and gene cluster identification of actinomycetes producing anti-mycobacterial compound

Genomic DNA was extracted using Quick-DNA™ HMW MagBead Kit (Zymo Research D6060) according to the manufacturer's instruction. A draft genome was produced using GridION X5 Mk1 equipped with FLO-MIN114 flow-cell and SQK-NBD114-24 sequencing kit (Oxford Nanopore Technologies). The base-calling was conducted using Guppy 6.3.8 with a High-accuracy model (HAC). Reads were assembled using Flye 2.9.1 [25] and polished by Medaka 1.7.2 [26]. Genome annotation was carried out using the Rapid Annotation with Subsystem Technology (RAST) server (https://rast.nmpdr.org) [27]. The biosynthetic gene clusters (BGCs) were identified from the draft genome using antiSMASH 7.0 (https://antismash.secondarymetabolites.org) [28].

## 3 Results

### 3.1 Identification of potent mycobacterium inhibitors by screening of actinomycetes extracts

**Fig. 1:**
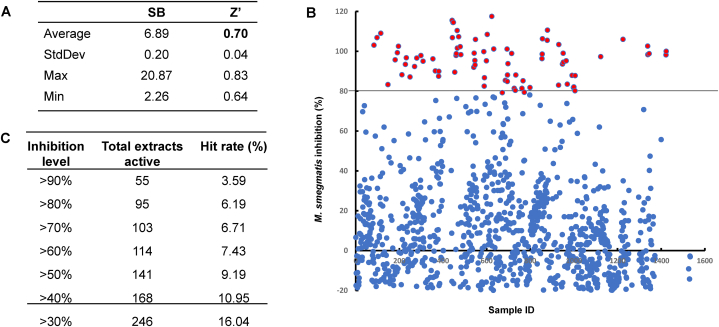
Performance metrics of actinomycetes extracts screening against *M. smegmatis*. Quality control values of all twenty 96-well assay plates in the primary screening of the extracts (1,534 extracts). The values of the S/B ratio (SB) and Z′ are shown **(A)**. A plot showing percentage inhibition of anti-mycobacterial activity by all extracts in the primary assay **(B)**. Inhibition level and hit rate calculation from primary screening **(C)**.

**Fig. 2:**
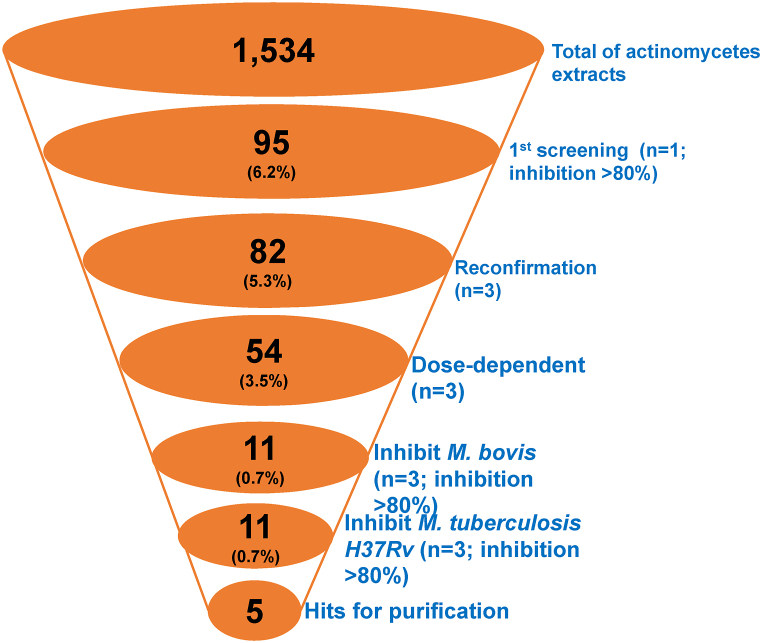
A screening cascade of actinomycetes extracts against *Mycobacterium*. The number of technical replicate assays is indicated (n). Individual steps of the cascade are described in the text.

We evaluated the growth inhibitory activity of 1,534 actinomycetes extracts against *M. smegmatis*, as illustrated in Fig. 1. The screening procedure employed a singular final extract concentration of 100 μg/mL. The assay was highly reliable based on the Z’ factor value of 0.7 (Fig. 1A) [19]. Utilizing a selection criterion of >80 % inhibition (Fig. 1B), we identified 95 primary hits, achieving an overall hit rate of 6.2 % (Fig. 1C). In the secondary confirmation screening, triplicate testing was adopted to eliminate potential reading errors stemming from the initial single-concentration screening. Consequently, the confirmation screening led to the confirmation of 82 hit extracts. Out of the initial 82 hits from the extracts, we narrowed it down to 54 extracts based on dose-dependent experiments. (Fig. 2). We selected extracts that exhibited linear inhibition across varying concentrations for the next phase of analysis.

Fifty-four confirmed hits were subjected to testing against *M. bovis*. Eleven out of 54 extracts exhibited >80 % inhibition against *M. bovis*. Subsequently, these eleven *M. bovis* hit extracts underwent assessment against *M. tuberculosis* pathogenic H37Rv strain. Remarkably, all eleven strains consistently displayed a good inhibitory effect against *M. tuberculosis*, at comparable levels to *M. smegmatis* and *M. bovis*. Among these, five extracts showing inhibition levels of ∼100 % were deemed particularly promising and therefore prioritized for purification. In this report, we described the characterization of only one of these potent extracts, while the remaining active extracts will be described elsewhere. A representative potent actinomycetes A612 strain was subsequently cultured on a large scale for purification of the active inhibitory compounds.

### 3.2 Characterization of the purified anti-mycobacterial compound

**Fig. 3:**
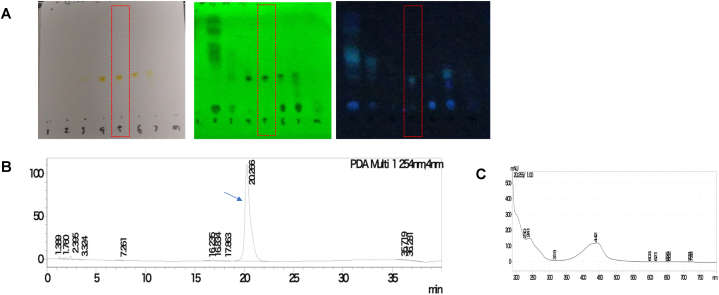
Evaluation of purification process and purity of active compound. The TLC pattern of the pure compound observed with cerium sulfate stain (right side), UV wavelength 254 nm (middle) and 366 nm (left) with eluent dichloromethane: methanol (20 : 1). A red bracket indicating the specific location of the targeted pure active compound. **(A).** The quantification of the purity of the active compound was performed using HPLC. The active peak is indicated by the blue arrow, and it exhibited a purity of 97.2 % **(B)**. UV visible spectrum absorbance of active compound after purification **(C)**.

**Fig. 4:**
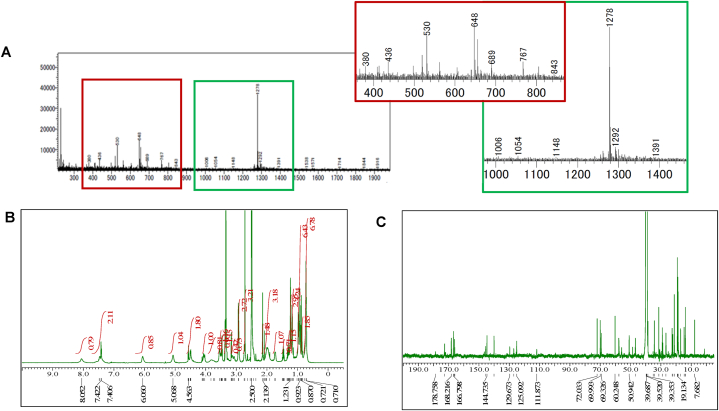
Mass Spectra **(A)**, 1H NMR **(B)** and 13C NMR **(C)** of purified compounds from strain A612.

From approximately 237.5 mg of crude extract, we collected 32.3 mg of pure compound after several purification steps. That compound was evaluated using TLC (Fig. 3A) and confirmed a singular peak indicating a purity of 97.2 % (Fig. 3B). The active compound, F5(23), was soluble in dichloromethane, chloroform, methanol, ethyl acetate and DMSO. The compound was orange in color, with the absorption maximum (*λ*_max_) at 241 and 442 nm (Fig. 3C), and the *m*/*z* value (M+) of 1278 **(**Fig. 4A**)**. The structure of the active compound was elucidated using ^1^H NMR **(**Fig.s. 4B), ^13^C NMR (Fig. 4C), and ESI-MS spectra. These findings and analyses provide a comprehensive characterization of the compound. A high-resolution ^1^H NMR spectrum was acquired at 500 MHz. Tetramethylsilane (TMS) served as the internal standard, and the solvent used was CD3OD. The resulting data are presented below: δ 8.05 (s, 1H), 7.48–7.41 (m, 2H), 6.06 (s, 1H), 5.07 (s, 1H), 4.57–4.49 (m, 2H), 4.07–4.03 (m, 1H), 3.83–3.71 (m, 1H), 3.54–3.50 (m, 1H), 3.47 (q, *J* = 5.4 Hz, 1H), 3.38–3.35 (m, 1H), 3.16 (d, *J* = 5.2 Hz, 0.5H), 3.14–3.06 (m, 1H), 2.94–2.92 (m, 3H), 2.72 (s, 3H), 2.14 (s, 1H), 2.07–1.91 (m, 3H), 1.74–1.72 (m, 1H), 1.49–1.44 (m, 1H), 1.34–1.27 (m, 1H), 1.23 (s, 3H), 1.18–1.14 (m, 3H), 1.02–0.91 (m, 6H), 0.89–0.84 (m, 2H), 0.78–0.71 (m, 7H). Based on characteristics and parameters collected (Fig. 4 and Fig. S1), the compound F5(23) has a molecular weight of approximately 1,278 g/mol and is similar to the actinomycin group, suggesting that F5(23) closely resembles actinomycin D.

### 3.3 Determination of IC_50_ for pure compound against mycobacterium cell

**Fig. 5:**
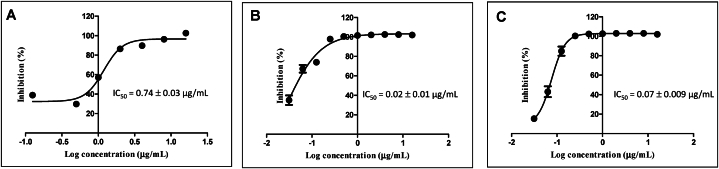
Dose-dependent inhibition for IC50 determination of active compound inhibited *M. smegmatis***(A)**, *M. bovis***(B)** and *M. tuberculosis***(C)**.

The antimicrobial activity of F5(23) was assessed against multiple mycobacterial species, *M. smegmatis*, *M. bovis*, and *M. tuberculosis* H37Rv. F5(23) exhibited an inhibitory effect against all three *Mycobacterium* species. Subsequently, the IC_50_ values of F5(23) against *M. smegmatis* (Fig. 5A), *M. bovis* (Fig. 5B) and *M. tuberculosis* (Fig. 5C) were determined to be 0.74, 0.02, and 0.07 μg/mL, respectively. It is noteworthy that F5(23) demonstrated high potency against *M. tuberculosis*, exhibiting approximately a 10-fold greater effect compared to rifampicin (the IC_50_ of rifampicin against *M. tuberculosis* H37Rv is 0.8 ± 0.12 μg/mL).

### 3.4 Antimicrobial spectrum activity of F5(23)

To explore the activity spectrum of F5(23), we conducted antimicrobial assays against a range of microorganisms, encompassing Gram-positive and Gram-negative bacteria as well as fungi (Table 1). Our findings revealed a distinct activity against specific strains of Gram-positive bacteria, while no inhibitory effects were observed against Gram-negative bacteria or fungi. These results indicate that F5(23) is classified as a narrow-spectrum antibiotic targeting Gram-positive bacteria.

**Table 1:** IC_50_ value of F5(23) compound and drug control (Rifampicin and Actinomycin D) against bacterial, fungi, and colorectal adenocarcinoma cell line (DLD-1).

Antagonist tested	IC50 (μg/mL)		
	F5(23)	Rifampicin	Actinomycin D
Gram-positive bacteria			
*Mycobacterium smegmatis*	0.74 ± 0.03	0.48 ± 0.07	0.78 ± 0.21
*Mycobacterium bovis* var BCG	0.02 ± 0.01	<0.03	0.8 ± 0.09
*Mycobacterium tuberculosis* H37Rv	0.07 ± 0.009	<0.01	0.8 ± 0.12
*Bacillus subtilis*	<0.3	<0.3	<0.3
*Staphylococcus aureus*	<0.3	<0.3	<0.3
**Gram-negative bacteria**			
*Salmonella enterica*	ND	NA	14.65 ± 1.22
*Escherichia coli*	ND	NA	ND
*Pseudomonas aeruginosa*	ND	NA	ND
**Fungal**			
*Candida albicans*	ND	NA	NA
*Saccharomyces cerevisiae*	ND	NA	NA
*Aspergillus niger*	ND	NA	NA
**Human cell line**			
DLD-I	>80	NA	NA
**Selectivity index (DLDI/*M. tuberculosis*)**	>1,100		

Note: ND, no inhibition detected in our maximum concentration tested (100 μg/mL) and considered as not toxic or no activity; NA, not applicable or not check in this experiment. The assays were carried out three times independently, and the results are shown as means ± SEM of triplicates.

### 3.5 Comparative activity of F5(23) and actinomycin D

NMR and MS analyses substantiated the structural resemblance of F5(23) compound to actinomycin D. To further elucidate this relationship, a comprehensive bioactivity comparison was conducted, utilizing the standard actinomycin D (Sigma, A1410) as a reference. F5(23) and standard actinomycin D exhibited almost similar trends of bioactivity (Table 1). However, F5(23) demonstrated a 10-fold increase in potency against *M. bovis* and *M. tuberculosis*, compared to actinomycin D. Conversely, F5(23) showed no inhibitory effect against *Salmonella enterica*, whereas actinomycin D demonstrated an IC_50_ value of 14.7 μg/mL. Further exploration focused on the inhibition of the enzymatic activity of *Mt*SK. F5(23) showed the IC_50_ value of 41.2 ± 5.2 μg/mL, two-fold lower compared to actinomycin D. Despite some differences in biological activity observed between F5(23) and actinomycin D, the chemical characterization, including NMR and MS analyses, strongly aligns with actinomycin group.

### 3.6 Identification of strain A612 producing F5(23) compound

**Fig. 6:**
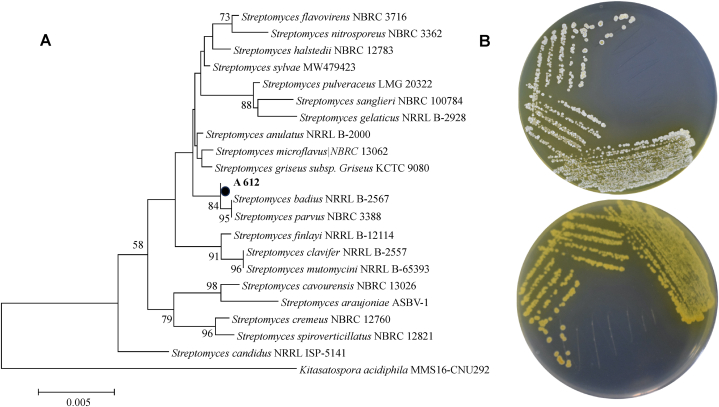
The phylogenetic tree and morphology of strain A612. Phylogenetic tree derived from 16S rRNA gene sequences of strain A612 and members of the genus Streptomyces, reconstructed with the neighbor-joining method. The 16S rRNA gene sequence of *Kitasatospora acidiphila* MMS16-CNU292 was used as the outgroup. Bootstrap values (>50 %) based on 1000 replicates are shown at branch nodes. Bar, 0.005 Knuc substitutions per nucleotide position **(A)**. The morphology of A612 on Yeast Starch Agar after incubation for 5 days. The presence of dissolved pigment in the agar medium was observed **(B)**.

F5(23)-producing A612 strain was characterized by a thorough comparison of its 16S rRNA gene sequence with sequences accessible in the GenBank repository, the National Center for Biotechnology Information (NCBI, USA). This analysis conclusively positioned A612 within the group of the *Streptomyces* genus. A612 strain exhibited a close kinship with two prominent members of the *Streptomyces* genus, *S. badius* and *S. parvus*, with >99 % similarity. The phylogenetic analysis by a neighbor-joining (NJ) method using the MEGA-X software, the constructed 16S rRNA gene phylogenetic tree further underscored its taxonomic affiliation (Fig. 6A). A612 strain displayed robust growth on the majority of the utilized culture media (data not shown). It formed colonies with colors ranging from white to yellow on YSB media and produced a yellow diffusible pigment observed on the reverse side (Fig. 6B). Microscopic examination revealed that this strain produced round to oval spores with a smooth surface.

### 3.7 Genomic features of the strain A612

**Fig. 7:**
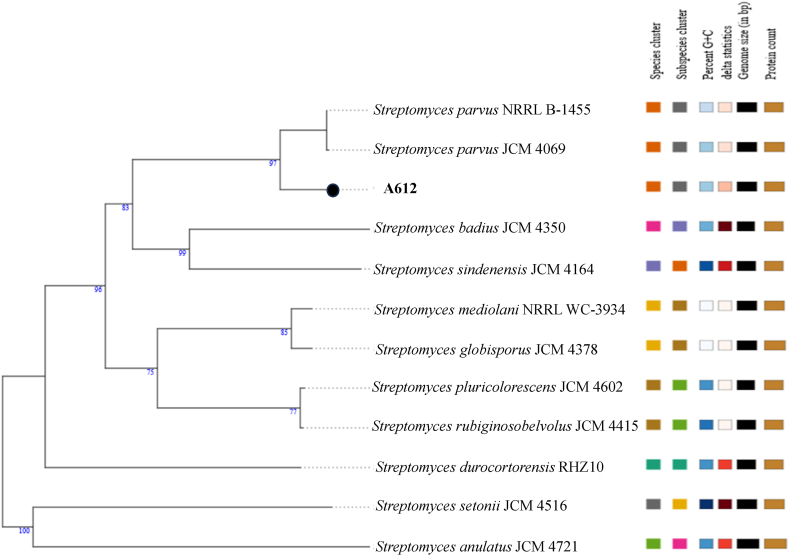
Whole-genome sequence-based tree from strain A612 and relative genome sequences. The branch lengths are scaled in terms of genome BLAST distance phylogeny (GBDP) distance formula d5 (Meier-Kolthoff and Göker, 2019). Numbers above branches are GBDP pseudo-bootstrap support values > 60 % from 100 replications.

**Fig. 8:**
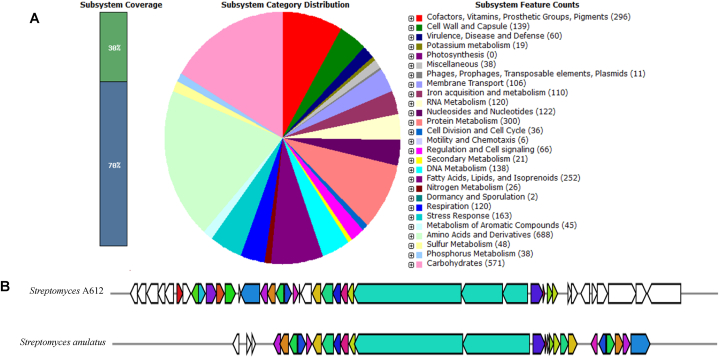
Distribution of subsystems from *Streptomyces* A612 strain annotated using rapid annotations subsystem technology (RAST) server **(A)** and A comparison of actinomycin gene clusters of *Streptomyces* A612 and *Streptomyces anulatus***(B)**. For figure A, the heat maps bar displayed in a grid, each row represents the similarity based on color and bar size by considering species cluster, subspecies cluster, percent G + C content, delta statistic, genome size, and protein count. Similar bar size and color indicated similar genome feature.

The genomic features of *Streptomyces* strain A612 were investigated through whole-genome sequencing. A612 possesses a total genome length of 8,179,066 base pairs (bps) and an average G + C content of 71.51 % (Table 2). Although phylogenetic analysis (Fig. 6A) suggested a close relationship among A612, *S. badius*, and *S. parvus*, a more comprehensive thorough phylogenomic considerations on various factors such as species cluster, subspecies cluster, percent G + C content, delta statistic, genome size, and protein count, conclusively places A612 within *S. parvus* species (Fig. 7). To determine the precise identity of A612 and its relationship with closely related species, we analyzed the average nucleotide identity (ANI) using the OrthoANIu web tool (https://www.ezbiocloud.net/tools/ani) [29]. A612 exhibited a genome-wide similarity of 98.61 % to *S. parvus* JCM 4069 and 93.74 % *to S. badius* JCM 4350. Thus, we proposed that A612 be classified as *Streptomyces parvus*. To further characterize its genetic potential, rapid annotation using Rapid Annotation Subsystems Technology (RAST) was used to identify 27 subsystems related to its metabolism (Fig. 8A).

**Table 2:** Characteristic of the A612 genome assembly and its annotation.

Feature	Value
Size (bp)	8,179,066
GC content (bp)	5,857,040
GC content (%)	71.61
Contig	1
N50	8,179,066
Coverage	108
Complete BUSCO (%)	90.54
CDC	7317^a^; 7324^b^; 7602^c^
rRNA	18^a,b,c^
tRNA	77^a^; 69^b^; 65^c^
tmRNA	1^a,b,c^

Note: Annotation for CDC, rRNA, tRNA and tmRNA was performed using various software. Subscribe a, b and c indicated for software Prokka v1.14.6, BaktaWeb 1.8.1 and RAST ver.2.0, respectively.

### 3.8 Prediction of the putative secondary metabolite's biosynthetic gene clusters

As *S. parvus* A.612 strain exhibited noteworthy antimicrobial activity against *Mycobacterium* species and other microbes, we employed genome analysis to predict Natural Product Biosynthetic Gene Clusters (BGCs) to investigate its potential to produce a range of natural products (NPs). AntiSMASH has been widely employed for the discovery of biosynthetic gene clusters in various organisms, including *Streptomyces* [30]. Computational genome analysis using antiSMASH 7.0 (Blin et al., 2021) revealed that the *Streptomyces* A.612 genome possesses the capacity to synthesize various categories of secondary metabolites. These categories encompass polyketides (facilitated by PKS or polyketide synthases), non-ribosomal peptides (NRPS or non-ribosomal peptide synthases), and bacteriocins. Leveraging the antiSMASH database, we detected a total of 40 BGCs within the genome of A.612. Remarkably, 24 clusters may represent potentially unknown or novel compounds while 16 remaining BGCs exhibited well-established functions (Table 3). A gene cluster responsible for actinomycin D synthesis was also identified, located within Cluster 33, regulated by Non-ribosomal Peptide Synthetase Biosynthetic Clusters (NRPS). This cluster spans approximately 66 kilobases and is localized in the genome between positions 7,444,697 and 7,510,757, composed of 41 genes (Fig. 8B). The putative actinomycin biosynthetic gene cluster of strain A.612 displayed an 85 % similarity to the actinomycin D gene cluster found in *Streptomyces anulatus*. In addition to this similarity, the A.612 actinomycin cluster contained additional biosynthetic genes, including pyridoxal-phosphate dependent enzymes, cytochrome P450, methyltransferase, kynureninase, a class I adenylate-forming enzyme family protein, HAD family hydrolase, and S8 family serine peptidase. The presence of these additional genes within this gene cluster, suggests that Streptomyces sp. A.612 may possess the potential to produce novel actinomycin analogs.

**Table 3:** Secondary metabolite gene clusters of *Streptomyces* sp. A612 as predicted by antiSMASH 7.0

Cluster	Type of secondary metabolite gene cluster	Similarity to known cluster	Similarity	Localization	
				from	to
1	T1PKS	stambomycin (A-D)	100 %	556	167,076
2	NRPS-like,T1PKS,NRPS	balhimycin	8 %	209,287	260,037
3	NRPS,T3PKS	tetronasin	11 %	324,183	429,423
4	thiopeptide,LAP	lactazole	33 %	434,127	462,175
5	melanin	melanin	100 %	472,892	483,374
6	RiPP-like	streptamidine	66 %	485,792	494,969
7	NRPS,T1PKS	valinomycin/montanastatin	13 %	517,373	565,474
8	RiPP-like	tetronasin	3 %	679,884	690,267
9	NRPS,T1PKS	SGR PTM Compound (b-d)	83 %	724,069	772,348
10	terpene	hopene	69 %	838,070	864,199
11	NRPS,PKS-like,NRPS-like	omnipeptin	15 %	1,012,829	1,090,870
12	terpene	2-methylisoborneol	100 %	1,345,914	1,366,200
13	NRPS	holomycin	92 %	1,557,163	1,598,813
14	NRPS,T1PKS	collismycin A	74 %	1,676,369	1,724,993
15	NRPS-independent-siderophore			1,940,963	1,953,995
16	terpene			2,371,078	2,390,100
17	ectoine	showdomycin	35 %	2,514,479	2,522,285
18	lanthipeptide-class-iii	AmfS	100 %	2,758,162	2,779,864
19	T1PKS,NRPS-like	enduracidin	8 %	2,867,518	2,920,449
20	lanthipeptide-class-iv	labyrinthopeptin (A1-A3)	40 %	3,529,029	3,551,911
21	lassopeptide	SRO15-2005	100 %	3,659,904	3,681,370
22	NRPS-like,arylpolyene, ladderane,NRPS	kitacinnamycin (A-F)	54 %	3,683,934	3,781,436
23	lanthipeptide-class-ii	chalcomycin A	9 %	3,976,338	4,002,517
24	lassopeptide	stlassin	75 %	4,733,922	4,756,535
25	NRPS	inthomycin B	18 %	4,766,421	4,820,642
26	NRPS-like			4,995,689	5,036,301
27	thiopeptide,LAP			5,117,258	5,149,792
28	NRPS-independent-siderophore	desferrioxamin B	100 %	5,559,638	5,571,416
29	lanthipeptide-class-iii,lanthipeptide-class-ii			5,633,119	5,664,303
30	ectoine	ectoine	100 %	6,616,807	6,627,205
31	terpene	steffimycin D	19 %	7,069,162	7,088,002
32	NRPS-like,NRPS	A83543A	8 %	7,194,017	7,251,962
33	other, NRPS	actinomycin D	85 %	7,444,697	7,510,757
34	T3PKS	naringenin	100 %	7,804,756	7,845,874
35	NRPS	retimycin A	13 %	7,846,742	7,895,720
36	NRP-metallophore,NRPS	coelichelin	81 %	7,900,501	7,959,156
37	NRPS	griseobactin	100 %	7,963,344	8,010,792
38	terpene	geosmin	100 %	8,042,320	8,064,533
39	butyrolactone	coelimycin P1	16 %	8,093,837	8,104,781
40	NRPS-like	nocathiacin	4 %	8,122,307	8,165,921

### 3.9 Molecular docking of actinomycin against some known essential *M. tuberculosis* target proteins and revalidating docking results through selected *M. tuberculosis* enzymes assay

**Fig. 9:**
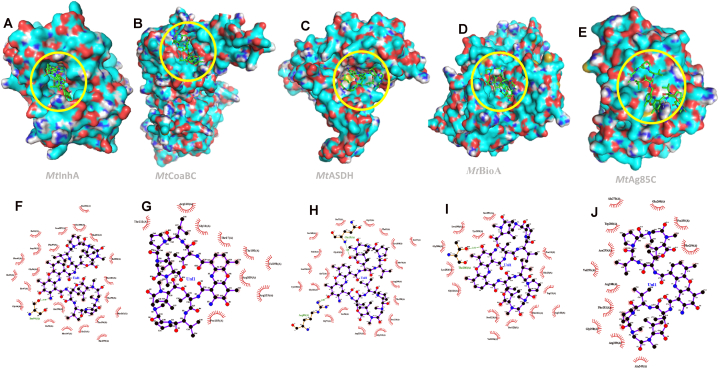
Docked compound F5(23) in the various *M. tuberculosis* protein. 3D (**A-E**) and 2D (**F-J**) interaction are presented. Enoyl reductase (**A and F**), Coenzyme A biosynthesis bifunctional protein (**B and G**), Aspartate-semialdehyde dehydrogenase (**C and H**), Adenosylmethionine-8-amino-7-oxononanoate aminotransferase (**D and I**) and Antigen 85 complex (**E and J**). The yellow circle highlights the specific position where the compound has been docked within the protein.

**Fig. 10:**
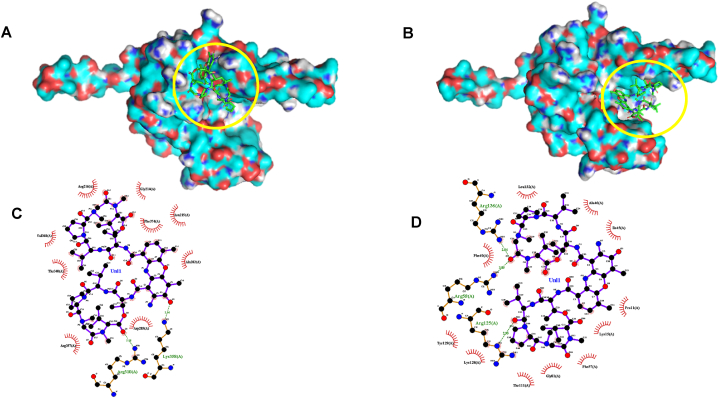
The docking of compound F5(23) into the *M. tuberculosis* shikimate kinase (*Mt*SK) enzyme. The 3D and 2D visualizations for two binding sites within *Mt*SK are presented. The first site (**A and C**) is where ATP typically binds naturally, and the second site (**B and D**) is where shikimate natively docks. The yellow circle is used to emphasize the precise location where the compound has been docked within the protein.

To better understand the possible mechanism of action of F5(23) against *M. tuberculosis*, a molecular docking study was conducted, focusing on 21 enzymes known to be anti-tubercular drug targets. Since F5(23) was identified as actinomycin D, we used its structure for docking studies. The calculated binding energies of the actinomycin D to 21 potential targets are shown in Table 4. The evaluation of the likeliness of protein-actinomycin D interaction is based on the criteria that the energy affinities are either equal to or lower than those of the native ligands. Among the 21 targets investigated, this compound appears to interact with multiple targets, including *Mt*InHA, *Mt*CoABC, *Mt*SK, *Mt*ASDH, *Mt*BioA, and *Mt*Ag85C. The intermolecular interactions of F5(23) were visualized (Fig. 9, Fig. 10A–D).

**Table 4:**
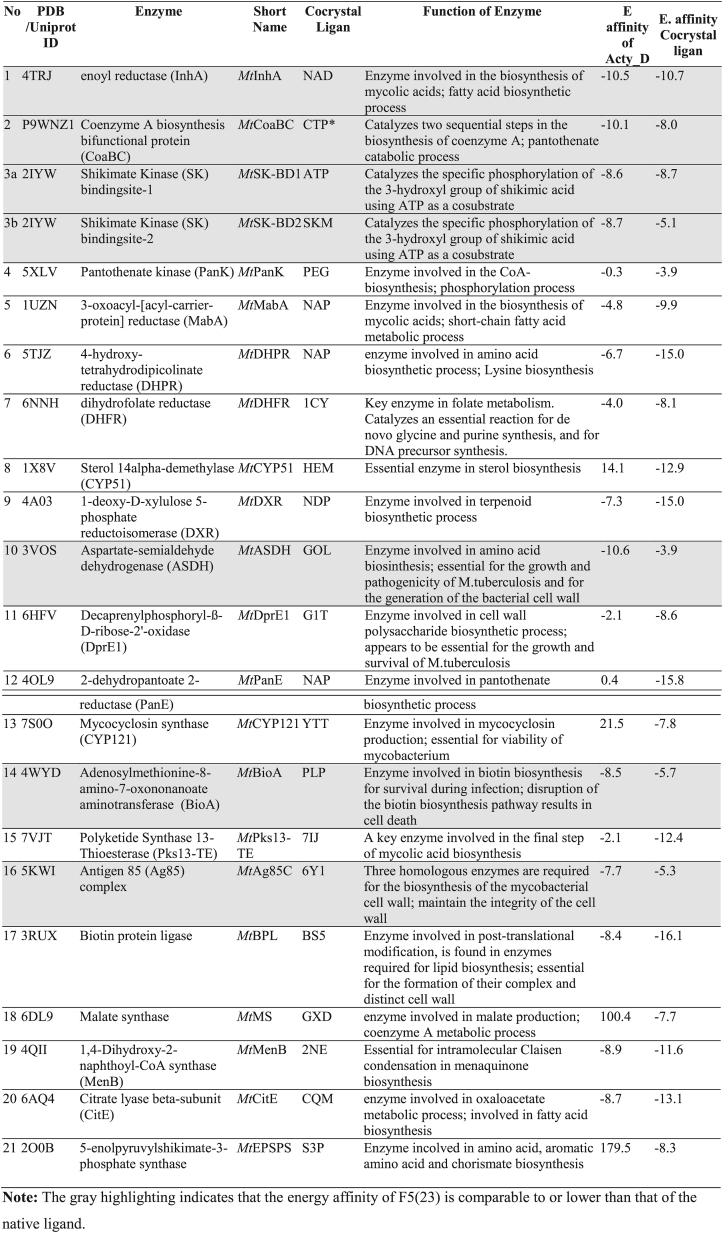
Results of molecular docking of compound F5(23).

Careful manual inspection of the interaction between actinomycin D and the 6 target proteins implicated by the docking study, prompted us to test whether actinomycin D inhibits *Mt*SK. The result indicated that actinomycin D exhibited relatively weak inhibitory activity against the recombinant enzyme *Mt*SK (Table 5). Shikimate kinase is an enzyme that catalyzes the ATP-dependent phosphorylation of shikimate to generate shikimate 3-phosphate. Upon docking, actinomycin D appears to occupy the ATP-binding pocket of *Mt*SK in a manner partially resembling its native ligand, ATP. Notably, hydrophobic interactions occur with Pro155, Arg153, Arg109, Val158, Thr150, Thr17, Gly12, Arg110, and Thr111 (Fig. 10A and C; Table S1). In contrast, among amino acid residues implicated for interactions with shikimate, only several residues are implicated to be engaged in hydrophobic interactions with actinomycin D: Gly81, Phe57, Ile45 and Phe49 (Fig. 10B and D; Fig. S2; Table S1).

**Table 5:** IC_50_ and *K*_i_ value of compound F5(23) and standard actinomycin D against selected *M. tuberculosis* recombinant enzymes.

Mycobacterium enzymes	Compound F5(23)		Standard actinomycin D	
	Inhibition Concentration (IC_50_)	inhibition constant (*K*i)	Inhibition Concentration (IC_50_)	inhibition constant (*K*i)
*Mt*SK	41.2 ± 5.2 μg/mL	58.0 ± 9.6 μg/mL	89.9 ± 6.7 μg/mL	119.2 ± 14.6 μg/mL
*Mt*CoABC	ND	ND	ND	ND
*Mt*EPSPS	ND	ND	ND	ND

Note: ND, no inhibition detected in our maximum concentration tested (100 μg/mL).

## 4 Discussion

The use of non-pathogenic *Mycobacteria* including *M. smegmatis*, instead of *M. tuberculosis* as surrogates, remains a subject of debate, mainly based on a limited success in TB drug discovery using non-pathogenic *M. smegmatis* strains [31,32]. However, the scarcity of academic institutions with biosafety level 3 (BSL-3) laboratories, particularly in low- and middle-income countries, along with the pathogenic nature and slow growth of *M. tuberculosis*, underscores the continued relevance of *M. smegmatis* as a tool in the early discovery phase, particularly in primary screening of libraries. The primary and confirmed hits obtained using *M. smegmatis* can be further validated using *M. tuberculosis* H37Rv strain. We took this approach and reconfirmed using *M. bovis* and, subsequently, *M. tuberculosis* H37Rv strain to ensure the translational potential of the identified microbial extracts.

A relatively high rate of hit reconfirmation of active actinomycetes extracts with *M. tuberculosis* H37Rv (20 %) has ensured the quality of our microbe and extract libraries. Furthermore, these results validated the utilization of *M. smegmatis* as a surrogate for TB screening. This rate is relatively low compared to other primary screenings conducted on chemical libraries [33]. One of the important observations in our study is that the inhibition profile of active extracts between *M. bovis* and *M. tuberculosis* demonstrated a remarkable similarity. This finding supports the potential of *M. bovis* as an ideal surrogate for *M. tuberculosis* on screening of at least *Actinomyces* culture broth libraries. Our data agree with the previous indication [33] that *M. smegmatis* falls short as a surrogate in comparison to *M. bovis* for anti-*M. tuberculosis* discovery.

The active compound F5(23) that we purified exhibits characteristics suggesting a close relationship to the actinomycin group, particularly resembling actinomycin D. Some minor discrepancies in the spectrum and the species specificity between F5(23) and standard actinomycin D remain unexplained. However, it is important to note that the chemical evidence we found strongly indicates that this compound is indeed actinomycin D. Actinomycins constitute a class of chromopeptide lactone antibiotics, with a total of 42 distinct actinomycin compounds having been isolated and characterized from various *Streptomyces* species [34,35]. Renowned for their use in clinical cancer treatments, actinomycins demonstrate remarkable antitumor efficacy [[36], [37], [38]]. Beyond their anticancer potential, actinomycins showcase the ability to impede certain viruses [39,40]. Additionally, these compounds often exhibit robust antimicrobial properties, which display notable effectiveness against tuberculosis [[41], [42], [43]].

Our genome analysis of the F5(23) producer A.612 revealed that this microbe has a gene cluster in the non-ribosomal peptide synthetase (NRPS). Similar to our result, recent advancements in genome research have unveiled the existence of the biosynthetic gene cluster (BGC) responsible for actinomycin D production in *S. chrysomallus*. This gene cluster spans a continuous stretch of 50 kb on a chromosome and comprises 28 biosynthetic genes [44]. A distinctive hallmark of actinomycins is the central phenoxazinone chromophore, which acts as a linkage between two pentapeptide lactones that are synthesized by the NRPS [45,46]. Earlier investigations in different species have also indicated that the regulation of actinomycin involves several genes, namely *acnW, acnR, acnU4,* and *acnO*. Additionally, a region of approximately 39.8 kb, housing 24 open reading frames (ORFs), has been identified as a potential coding site for actinomycin D biosynthesis in *S. costaricanus*. A proposed model for the assembly of actinomycins has also been documented in this species, with particular emphasis on the indispensable role of *acnF* as the pivotal gene in actinomycin D biosynthesis [47]. The gene encoding the tryptophan 2,3-dioxygenase family protein was also identified in strain A612, showing a 74–75 % amino acid similarity compared to its counterparts in *S. costaricanus and S. anulatus*. In general, if we compare to the closely related actinomycin gene cluster in *S. anulatus* (HM038106.1), the actinomycin gene cluster in Streptomyces A612 exhibits a nucleotide and amino acid with similarity of 77.2 % and 70.1 %, respectively. Strain A612 possesses three non-ribosomal peptide synthetase (NRPS) genes with a combined length of 20.5 kB.

To date, the specific mode of action of actinomycin D remains unclear. In cancer cells, the biological activities of actinomycin D are thought to involve its interaction with DNA, primarily through intercalation into double-stranded DNA, with a particular affinity for binding to CGG triplet repeat sequences [48]. This interaction can disrupt processes such as replication and transcription [49]. However, the precise mechanism by which actinomycin D inhibits *M. tuberculosis* remains to be understood. Prior research has indicated that actinomycin D has a weak inhibitory effect on *M. tuberculosis* pantothenate synthetase [50,51]. Although the activity is weak, other docking results have confirmed this inhibition based on lower energy binding scores. Additionally, it was recently suggested that actinomycin potentially interacts with *M. tuberculosis* protein kinase B (*Mt*PknB) [52]. *Mt*PknB plays a vital role in multiple biochemical pathways in *Mycobacteria* and is essential for growth and survival within the hosts [53]. However, validation of this protein as the target of actinomycin D has not yet been performed.

Due to the propensity of actinomycin D to interact with multiple targets, we embarked on docking studies to predict potential targets leading to growth inhibition. Our docking results highlighted that actinomycin D has the potential to interact with at least 6 out of the 21 protein targets we examined. Remarkably, we have experimentally confirmed that actinomycin D is capable of inhibiting *M. tuberculosis* shikimate kinase (*Mt*SK*) in vitro*, albeit with relatively weak activity (IC_50_ = 41.2 μg/mL). We also calculated the inhibition constant (*K*_i_) of this compound and the obtained *K*_i_ was slightly higher than IC_50_ value (*K*_i_ = 56.8 μg/mL), which may indicate that actinomycin D is a noncompetitive inhibitor of *Mt*SK. As previously reported, *Mt*SK is an indispensable enzyme in various pathogenic bacteria, including *M. tuberculosis*, and is absent in humans [54], making it an ideal drug target.

*Mt*SK contains a short conserved sequence motif, GXXXXGKT/S [55]. The native ligand, ATP, or its analog predominantly interacts with the P-loop residues: Gly12, Gly14, Lys15, Ser16, and Thr17. Additionally, ATP shows interactions with Arg117, Arg110, and Pro155. The other native ligand of *M*tSK, shikimate, interacts with the binding pocket residues (Ile45, Phe49, and Phe57), the P-loop, as well as three hydrophobic residues (Pro11, Pro118, and Leu119), and three highly conserved residues (Gly79, Gly80, and Gly81). Shikimate also forms interactions with the guanidium groups of Arg58 and Arg136 [56]. Our docking results revealed that this compound interacts with Gly12, Thr17, Arg109, and Pro155, which are similar to the amino acids interacting with ATP. Furthermore, actinomycin D also interacts with Arg58 and Gly81, coinciding with the positions of the carboxylate group of shikimate when bound to *Mt*SK. However, only two essential amino acid residues are shared in the interaction between actinomycin D and shikimate. Hence, we propose that actinomycin D likely acts as a partially competitive inhibitor, primarily competing with the ATP substrate. Our findings are corroborated by prior research, which demonstrated that actinomycin D has inhibitory effects on pantothenate synthetase [50,51]. Additionally, it was observed that actinomycin D also affects protein kinase PnKB [52], an enzyme reliant on ATP for its activity.

## 5 Conclusions

We conducted screening of 1,534 actinomycete culture extracts for growth inhibition against *M. smegmatis*, leading to the identification of extracts that exhibited over 80 % growth inhibition. The overall hit rate from this screening was 6.2 %. We subsequently validated the efficacy of these extracts against *M. bovis* and *M. tuberculosis*, and ultimately isolated an active compound from a single potent extract. This compound, which displayed potent inhibition against all three mycobacterial species, was structurally elucidated to be actinomycin D. Moreover, we identified the producer of this active compound as *Streptomyces parvus* based on 16S rRNA sequence analysis, phylogenetic reconstruction, and whole genome sequencing. This *S. parvus* A.612 strain possesses a complete genome size of 8,179,066 base pairs and boasts 40 biosynthetic gene clusters capable of producing various known and potentially unknown compounds. Among these clusters, we found an NRPS gene cluster that shares an 85 % similarity to the one responsible for actinomycin D production in other species. Molecular docking studies indicate potential target enzymes in *M. tuberculosis*. Six potential protein targets for actinomycin D were identified, with *Mt*SK being one of the most likely targets. Inhibition of the recombinant *Mt*SK confirmed that the identified active compound showed an IC_50_ value of 41.2 μg/mL against *Mt*SK. Since this IC_50_ value was significantly higher than that observed for cells, *Mt*SK is unlikely the sole target of actinomycin. Further investigation is still needed to clarify the mechanisms of action of actinomycins against *M. tuberculosis.*

## Data availability statement

All data generated in this study has been included in this article. The genome raw data are available on the NCBI Sequence under the accession number CP159802.  

## CRediT authorship contribution statement

**Arif Nurkanto:** Writing – review & editing, Writing – original draft, Supervision, Methodology, Investigation, Funding acquisition, Formal analysis, Data curation, Conceptualization. **Masrukhin:** Methodology, Investigation, Data curation. **Joseph Calvin Erdian Tampubolon:** Data curation. **Muhammad Farrel Ewaldo:** Data curation. **Ade Lia Putri:** Investigation, Data curation. **Shanti Ratnakomala:** Validation, Methodology. **Ruby Setiawan:** Visualization, Software. **Ahmad Fathoni:** Visualization, Software, Methodology. **Kartika Dyah Palupi:** Data curation. **Yulia Rahmawati:** Data curation. **Danang Waluyo:** Project administration, Methodology. **Erwahyuni Endang Prabandari:** Methodology, Data curation. **Sri Pujiyanto:** Investigation. **Yuji Sumii:** Methodology, Investigation, Formal analysis. **Andria Agusta:** Supervision, Methodology, Conceptualization. **Norio Shibata:** Validation, Supervision. **Sohkichi Matsumoto:** Writing – review & editing, Supervision, Methodology. **Tomoyoshi Nozaki:** Writing – review & editing, Writing – original draft, Supervision, Funding acquisition, Conceptualization.

## Declaration of competing interest

The authors declare the following financial interests/personal relationships which may be considered as potential competing interests:Arif Nurkanto and Tomoyoshi Nozaki reports financial support, administrative support, article publishing charges, equipment, drugs, or supplies, and travel were provided by Rispro 10.13039/501100014538LPDP Indonesia, 10.13039/501100009037Science and Technology Research Partnership for Sustainable Development (10.13039/501100009037SATREPS) from the 10.13039/100009619Japan Agency for Medical Research and Development (10.13039/100009619AMED) and 10.13039/501100004532Japan International Cooperation Agency (10.13039/501100004532JICA). If there are other authors, they declare that they have no known competing financial interests or personal relationships that could have appeared to influence the work reported in this paper.
