# Supplementary material for: Exploring Indonesian actinomycete extracts for anti-tubercular compounds: Integrating inhibition assessment, genomic analysis, and prediction of its target by molecular docking
Source: Heliyon. 2024 Aug 4;10(15):e35648. doi: 10.1016/j.heliyon.2024.e35648 (PMC11336835; doi:10.1016/j.heliyon.2024.e35648)
Supplement: Multimedia component 1 [file mmc1.pptx]

## Slide 1
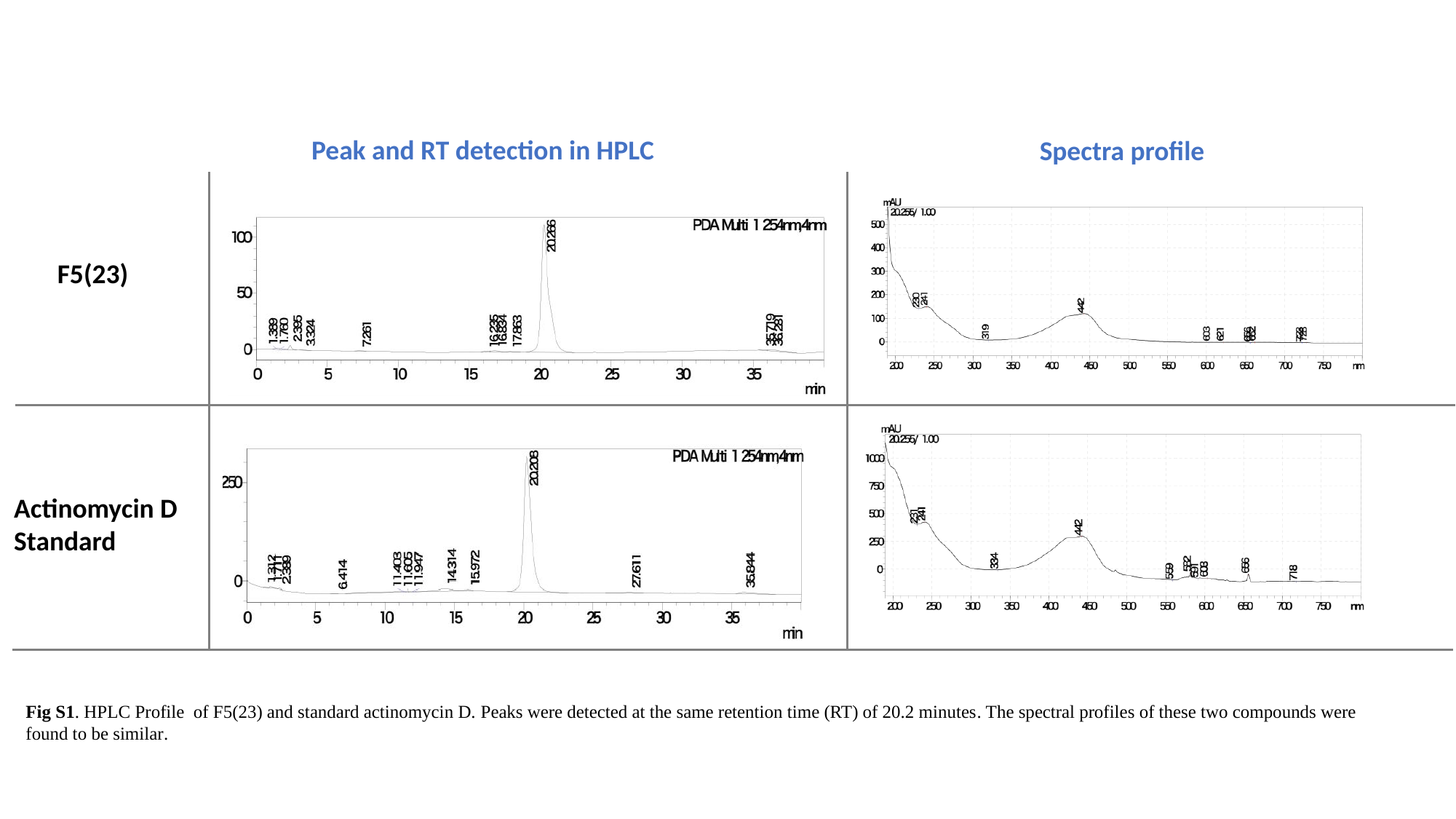

Peak and RT detection in HPLC
Spectra profile
F5(23)
Actinomycin D Standard
Fig S1. HPLC Profile of F5(23) and standard actinomycin D. Peaks were detected at the same retention time (RT) of 20.2 minutes. The spectral profiles of these two compounds were found to be similar.

## Slide 2
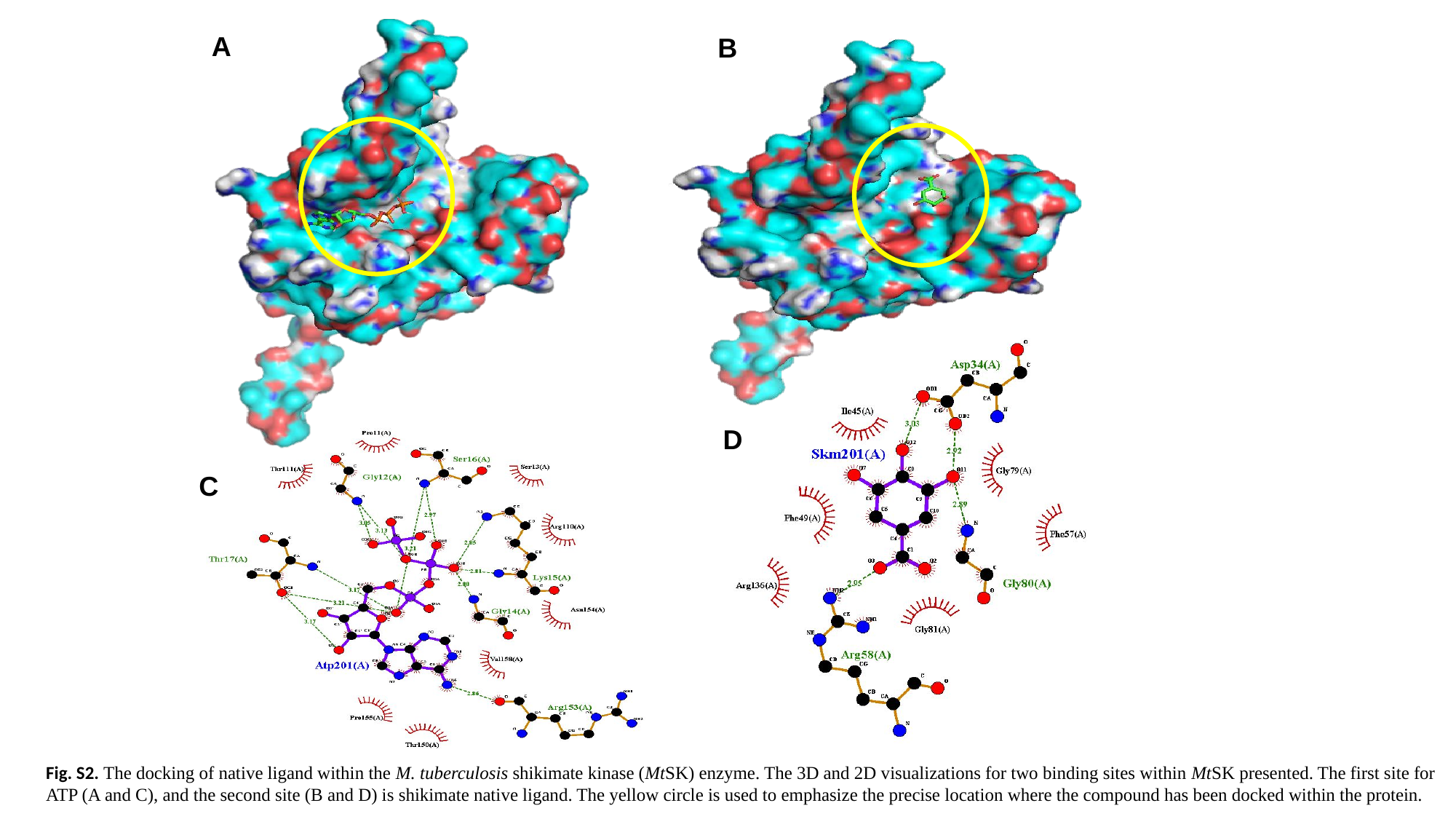

A
B
D
C
Fig. S2. The docking of native ligand within the M. tuberculosis shikimate kinase (MtSK) enzyme. The 3D and 2D visualizations for two binding sites within MtSK presented. The first site for ATP (A and C), and the second site (B and D) is shikimate native ligand. The yellow circle is used to emphasize the precise location where the compound has been docked within the protein.

## Slide 3
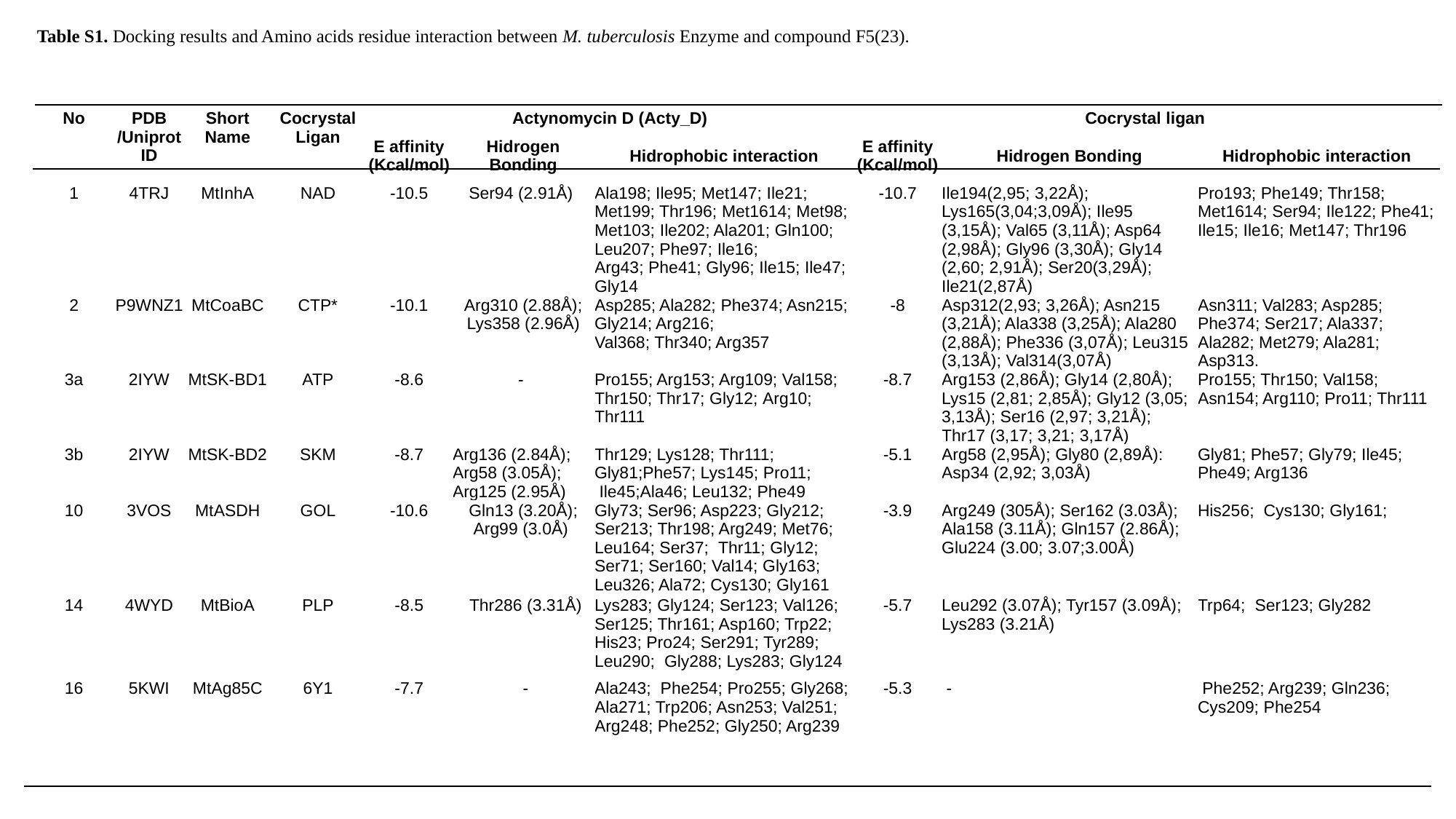

Table S1. Docking results and Amino acids residue interaction between M. tuberculosis Enzyme and compound F5(23).
| No | PDB /Uniprot ID | Short Name | Cocrystal Ligan | Actynomycin D (Acty\_D) | | | Cocrystal ligan | | |
| --- | --- | --- | --- | --- | --- | --- | --- | --- | --- |
| | | | | E affinity (Kcal/mol) | Hidrogen Bonding | Hidrophobic interaction | E affinity (Kcal/mol) | Hidrogen Bonding | Hidrophobic interaction |
| 1 | 4TRJ | MtInhA | NAD | -10.5 | Ser94 (2.91Å) | Ala198; Ile95; Met147; Ile21; Met199; Thr196; Met1614; Met98; Met103; Ile202; Ala201; Gln100; Leu207; Phe97; Ile16; Arg43; Phe41; Gly96; Ile15; Ile47; Gly14 | -10.7 | Ile194(2,95; 3,22Å); Lys165(3,04;3,09Å); Ile95 (3,15Å); Val65 (3,11Å); Asp64 (2,98Å); Gly96 (3,30Å); Gly14 (2,60; 2,91Å); Ser20(3,29Å); Ile21(2,87Å) | Pro193; Phe149; Thr158; Met1614; Ser94; Ile122; Phe41; Ile15; Ile16; Met147; Thr196 |
| 2 | P9WNZ1 | MtCoaBC | CTP\* | -10.1 | Arg310 (2.88Å); Lys358 (2.96Å) | Asp285; Ala282; Phe374; Asn215; Gly214; Arg216; Val368; Thr340; Arg357 | -8 | Asp312(2,93; 3,26Å); Asn215 (3,21Å); Ala338 (3,25Å); Ala280 (2,88Å); Phe336 (3,07Å); Leu315 (3,13Å); Val314(3,07Å) | Asn311; Val283; Asp285; Phe374; Ser217; Ala337; Ala282; Met279; Ala281; Asp313. |
| 3a | 2IYW | MtSK-BD1 | ATP | -8.6 | - | Pro155; Arg153; Arg109; Val158; Thr150; Thr17; Gly12; Arg10; Thr111 | -8.7 | Arg153 (2,86Å); Gly14 (2,80Å); Lys15 (2,81; 2,85Å); Gly12 (3,05; 3,13Å); Ser16 (2,97; 3,21Å); Thr17 (3,17; 3,21; 3,17Å) | Pro155; Thr150; Val158; Asn154; Arg110; Pro11; Thr111 |
| 3b | 2IYW | MtSK-BD2 | SKM | -8.7 | Arg136 (2.84Å); Arg58 (3.05Å); Arg125 (2.95Å) | Thr129; Lys128; Thr111; Gly81;Phe57; Lys145; Pro11;  Ile45;Ala46; Leu132; Phe49 | -5.1 | Arg58 (2,95Å); Gly80 (2,89Å): Asp34 (2,92; 3,03Å) | Gly81; Phe57; Gly79; Ile45; Phe49; Arg136 |
| 10 | 3VOS | MtASDH | GOL | -10.6 | Gln13 (3.20Å); Arg99 (3.0Å) | Gly73; Ser96; Asp223; Gly212; Ser213; Thr198; Arg249; Met76; Leu164; Ser37;  Thr11; Gly12; Ser71; Ser160; Val14; Gly163; Leu326; Ala72; Cys130; Gly161 | -3.9 | Arg249 (305Å); Ser162 (3.03Å); Ala158 (3.11Å); Gln157 (2.86Å); Glu224 (3.00; 3.07;3.00Å) | His256; Cys130; Gly161; |
| 14 | 4WYD | MtBioA | PLP | -8.5 | Thr286 (3.31Å) | Lys283; Gly124; Ser123; Val126; Ser125; Thr161; Asp160; Trp22; His23; Pro24; Ser291; Tyr289; Leu290;  Gly288; Lys283; Gly124 | -5.7 | Leu292 (3.07Å); Tyr157 (3.09Å); Lys283 (3.21Å) | Trp64;  Ser123; Gly282 |
| 16 | 5KWI | MtAg85C | 6Y1 | -7.7 | - | Ala243;  Phe254; Pro255; Gly268; Ala271; Trp206; Asn253; Val251; Arg248; Phe252; Gly250; Arg239 | -5.3 | - | Phe252; Arg239; Gln236; Cys209; Phe254 |
